# Characterisation of mental health conditions in social media using Informed Deep Learning

**DOI:** 10.1038/srep45141

**Published:** 2017-03-22

**Authors:** George Gkotsis, Anika Oellrich, Sumithra Velupillai, Maria Liakata, Tim J. P. Hubbard, Richard J. B. Dobson, Rina Dutta

**Affiliations:** 1 King’s College London, IoPPN, London, SE5 8AF, UK; 2 School of Computer Science and Communication, KTH, Stockholm; 3 Department of Computer Science, University of Warwick, Coventry; 4 King’s College London, Department of Medical & Molecular Genetics, London, SE1 9RT; 5 Farr Institute of Health Informatics Research, UCL Institute of Health Informatics, University College London, London, WC1E 6BT, UK

## Abstract

The number of people affected by mental illness is on the increase and with it the burden on health and social care use, as well as the loss of both productivity and quality-adjusted life-years. Natural language processing of electronic health records is increasingly used to study mental health conditions and risk behaviours on a large scale. However, narrative notes written by clinicians do not capture first-hand the patients’ own experiences, and only record cross-sectional, professional impressions at the point of care. Social media platforms have become a source of ‘in the moment’ daily exchange, with topics including well-being and mental health. In this study, we analysed posts from the social media platform Reddit and developed classifiers to recognise and classify posts related to mental illness according to 11 disorder themes. Using a neural network and deep learning approach, we could automatically recognise mental illness-related posts in our balenced dataset with an accuracy of 91.08% and select the correct theme with a weighted average accuracy of 71.37%. We believe that these results are a first step in developing methods to characterise large amounts of user-generated content that could support content curation and targeted interventions.

Mental and substance use disorders are the leading cause of years lived with disability worldwide and in 2010 accounted for 7.4% of years of productive life lost due to disability^1^. Natural language processing of electronic health records (EHRs) is increasingly being used to study mental illness^2^ and risk behaviours in much closer detail than previously^3^. However, narrative notes are written by clinicians who record those positive findings and relevant negatives that guide their subsequent diagnosis and treatment plan for the patient^4^. Although EHRs allow clinicians to synthesise disparate facts making them interpretable by other clinicians, they do not “paint a full picture” of the patient experience of a mental health problem, particularly as patients may answer interview questions in a manner that they perceive will be viewed favourably by their clinician. Moreover, as patient records are only written based on meetings with their healthcare provider, critical changes in patient behaviour and wellbeing may not be recognised either immediately or at all due to a time delay in reporting, thus preventing certain real time interventions.

Social media is changing the way people self-identify as suffering from a disorder and how they communicate with others with similar experiences, often asking about side effects from treatments, or sharing coping skills, and thereby feeling less isolation or stigma. Studying popular social media platforms such as Twitter and Reddit^5^,^6^ holds the key to understanding what concerns patients (rather than clinicians) most. Furthermore, this type of large-scale user-generated content offers the opportunity to understand mechanisms underlying mental health conditions at an unprecedented level. For instance, studies of children and adolescents have already shown that high daily use of social networking sites may be independently associated with poor self-rating of mental health and experiences of higher levels of psychological distress and suicidal ideation^7^.

Following on from our initial study^8^, here we present the second phase of our research on classifying user-generated content from Reddit that is related to different types of mental health conditions. We aimed to study the most epidemiologically prevalent and clinically burdensome mental health conditions: depression, bipolar disorder, anxiety disorders, schizophrenia, as described in the study by Whiteford *et al*.^1^. As in this most recent global clinical study, we also studied drug use disorders: general addiction, opiate addiction and alcoholism separately. With as many as 80% of suicide deaths attributable to mental and substance use disorders it was also relevant to study both suicide and self-harm behaviour. We captured pervasive developmental disorders by studying autism and regarded it as important to also include borderline personality disorder as an important example of a group for whom sparse global epidemiological data is available^1^ but who form a large, and active, online community.

In this paper, we address the problem of automated classification of mental health-related content on a social media platform. Our long-term goal is to not only aid clinical researchers, epidemiologists and policy makers in understanding and addressing communications on social media, but also to provide support for people suffering from mental illness. Here, we propose an approach to automatically identify Reddit posts related to mental health (binary classification) and then classify mental health-related posts according to theme-based subreddit groupings (multiclass classification) using deep learning techniques. Accompanied with manual content and topic analysis, we employ a theme-based approach to this problem, and compare results with alternative machine learning baselines. With Reddit providing monitoring and support through volunteers, being able to classify posts according to themes could enable alerting moderators by reposting posts to relevant communication threads if they have not been posted there yet. We also believe that the results and the methodology presented here is useful to gain a deeper understanding and to characterise language and content in these types of forums, and could be the first step in the development of targeted interventions using social media.

## Results

The basis of this study relies on the analysis of data extracted from Reddit. Reddit is a social media platform with over 234 million users (https://about.reddit.com/advertise/, accessed November 2016) that communicate in topic-specific communities, called subreddits. A report issued by PewResearchCenter showed that 6% of adult internet users frequently visit Reddit, and men are twice as likely than women to access Reddit contents^9^. Posts are not character-limited and users can freely express their thoughts in as many words and in as much detail as they wish. Subreddits can be created by users and there is no restriction on this, even when a similar subreddit already exists. As a consequence, there may be several subreddits discussing a specific mental health condition.

In order to assess whether or not posts are relevant to one mental health condition, and which health condition a relevant post belongs to, we developed an approach that combines manual assessment steps with automated topic detection and the application of deep learning classification of Reddit data.

**Figure 1:**
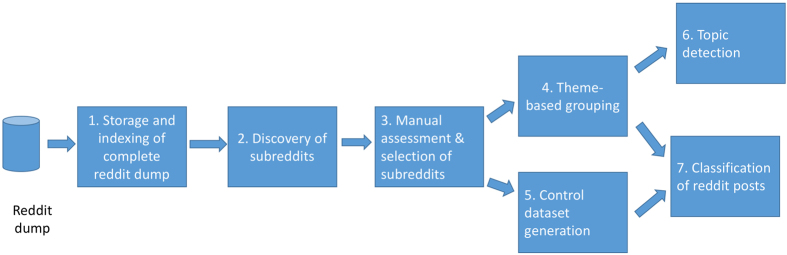
Overall workflow of our approach.

The general steps in our study are illustrated in Fig. 1 and further explained in the methods section. The first step includes the storage and indexing of the complete Reddit dataset (1). From this set, a semi-supervised discovery of relevant, mental health-related subreddits is applied (2). These subreddits are further analysed through a manual assessment step and the final list of subreddits is selected (3). The fourth step includes the grouping of subreddits that share the same mental health-related theme (4). The preparation of a control dataset is then performed (5), to be used for the binary classification task of posts as containing mental health-related content or not. The final steps include topic detection applied on our themes for confirmation of relevant content (6) and the two tasks of content (binary and multiclass) classification (7).

### Manual analysis and characterisation of subreddits

A manual content analysis was performed independently by two researchers. The aim was to reach a conclusion as to whether the post content reflects the mental health condition it relates to, i.e. the name of the subreddit. Ten random samples from each of the 16 subreddits (see methods section about the selection of the subreddits) were extracted, resulting in a set (gold standard) of mental health-related *themes*.

This manual analysis revealed that the post content in the majority of the subreddits was relevant to the mental health condition its title suggests. Most commonly, the posts were introduced by a descriptive section where the author of the post gave some context to the reason for writing, followed by a request for advice or help. The descriptive section typically contained highly relevant terms and condition-specific content.

One subreddit related to neurodevelopmental disorders (*Aspergers*) was deemed to be mainly distantly related to the condition itself, as a large proportion of posts (6/10) were not directly condition-related (e.g. requesting advice about recently diagnosed family members without details about the condition). Therefore, this was the only subreddit where the assumption that a subreddit post content is characteristic of its mental health condition did not hold, and it was excluded from the gold standard.

For the remaining 15 subreddits, the conclusion was that the post content was indeed characteristic of the intended mental health condition. Seven of these could be grouped into three mental health condition themes, resulting in a total of 11 themes (see Table 1).

The language in each of the resulting mental illness themes often contained specific references related to symptoms, treatments or experiences. The *depression* subreddit (by far the largest in terms of size: 42% of the total mental health related posts) was most disparate. For instance, it was common to find descriptions of situations and events experienced by post authors who were not necessarily diagnosed with depression, but where the user was saddened or negatively affected by some circumstance and used the word depressed colloquially rather than in a medical sense.

The themes related to self harming and suicide (*selfharm* and *SuicideWatch*) often contained explicit references to actions and thoughts, highly relevant to the condition. The drug use disorder themes (*opiates, cripplingalcoholism* and *addiction*) typically contained mentions of specific substances, medications and drugs, in combination with requests for help or advise (e.g. experiences with changing or trying specific drugs). Posts in the *cripplingalcoholism* subreddit were often more implicit, with a language use more indicative of being under the influence, rather than explicitly listing condition-related content. The only other subreddit related to neurodevelopmental disorders (*autism*) did, unlike the content in the *Aspergers* subreddit, predominantly contain disease-related descriptions about family members or close relatives along with questions about others’ experiences or requests for help related to the condition.

### Topic detection on mental health-related themes

As the manual characterisation was conducted only on a small set of posts for each of the subreddits, we added an additional assessment step of the generated themes using topic modelling. Topic modelling approaches use probability distributions of words to determine the topics that are represented in a collection of textual data. In this context, a topic is a collection of words that are assumed to be semantically related. This means that if we are able to extract topics that are closely related to the themes that we were hoping to represent, it is likely that the grouping of the themes is correct. Furthermore, the topics in themselves can provide some insights into what it is that patients and/or carers are most concerned about when facing a mental health condition.

The topic models we obtained for the 11 themes described in the previous section showed high relevance between the content of the posts and the particular mental health condition theme they had been assigned to, in line with the conclusions of the manual assessment. The entire list of topic models can be found in our supplement. Looking across the topic models of all the themes, we can see that there are various aspects of mental illnesses covered: symptoms, medications, potential side effects, treatment alternatives and impacts/consequences on life. For example, one of the topics formed for the *anxiety* theme (see topic 9, supplement) contains words such as ‘panic’, ‘attack’, ‘feel’, ‘like’, and ‘heart’. Anxiety sufferers in particular often see their general practitioner (GP) because they assume they have had a heart attack rather than suffering from anxiety. Furthermore, palpitations are a common symptom of anxiety, especially when panic attacks are present^10^. Similarly, one of the topics related to the bipolar theme (see topic 4, supplement) contains words like ‘lamictal’, ‘seroquel’, ‘lithium’, ‘take’, ‘meds’ and ‘taking’. In addition to words indicating the existence of a prescription for the medication, all the medications mentioned are used as mood stabilisers in the treatment of bipolar disorder.

In our manual analysis, two of the themes were highlighted as being less homogenous (*depression*) or diverging from the rest of the themes (*cripplingalcoholism*). However, in the topic analysis, we can still see clusters forming that are relevant to both conditions and the nature of the subreddit. For example, one of the clusters (see topic 5, supplement) for *depression* includes the words ‘don’t’, ‘job’, ‘money’, ‘pay’, ‘live’ and ‘life’, which could allude to the fact that people suffering from depression have difficulties keeping up with the responsibilities of a job, which may result in loss of income and the creation of obstacles in life^11^. The *cripplingalcoholism* theme has e.g. a cluster (see topic 2, supplement) containing words such as ‘like’, ‘cheers’, ‘drinking’, ‘day’, ‘happy’ and ’drink’, which suggests a glorifying aspect to being alcohol-dependent. In fact, the subreddit itself tries to involve not only those who want to defeat their addiction but also those who are content with possessing this addiction. The aforementioned cluster clearly shows evidence for the latter group of posters.

### Classification of mental health content

The process of automatically classifying Reddit posts was separated into two individual tasks. The first task was to classify the posts as being either mental health related or not (binary classification). The second task focussed on the classification of the manually defined and automatically evaluated themes, to determine which mental health theme the post belongs to (multiclass (n = 11) classification). Results presented below predominantly concern the output of the Convolutional Neural Network (CNN) based approach, since this consistently yielded the best prediction. For the training and evaluation of all classifiers, we used identical, randomly extracted, 80–20 training-test split across the entire dataset, and we report on the results concerning the test dataset. Further details about the classifiers and settings are presented in the Methods section.

#### Binary classification of mental from non-mental health related content

The best approach (CNN) yields an accuracy of 91.08% for an almost balanced dataset: 52% of the overall posts are related to mental health (see Tables 2 and 3). The other classifiers’ results were lower, with the Feed Forward (FF) being marginally worse (90.79%). The text was preprocessed identically for all four classifiers. A linear classifier achieves 85.84% accuracy whereas an SVM-based classifier achieves 86% accuracy.

#### Multiclass classification of mental health themes

**Figure 2:**
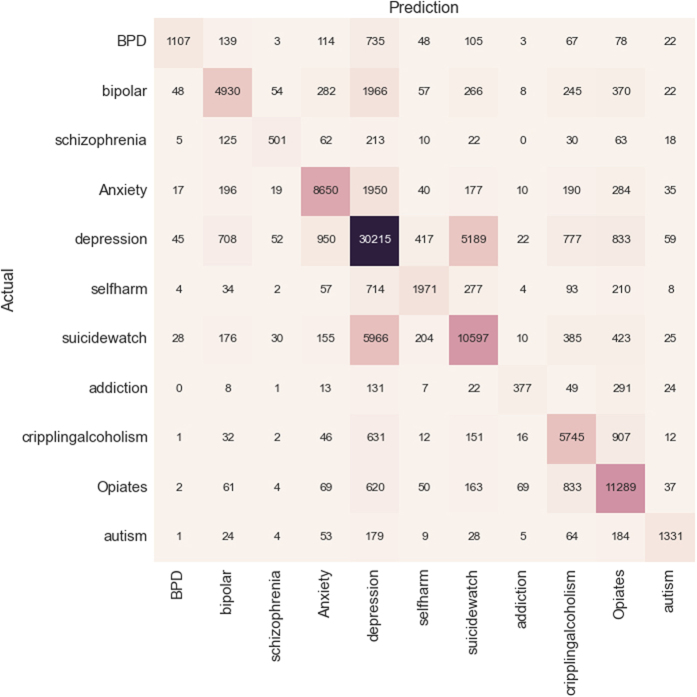
Multiclass classification confusion matrix using a Convolutional Neural Network (CNN) classifier.

The second set of results relate to the task of classifying mental health-related posts into one of the 11 themes. Figure 2 shows the resulting confusion matrix using a CNN approach. The figure shows that the left-to-right diagonal holds high values, and in the majority of the cases, the Reddit posts are classified correctly.

Table 4 presents the evaluation metrics (precision, recall and F-Measure) for each individual theme as well as the weighted average when using the CNN-based approach. The table shows that all results remain high, resulting in an overall weighted average of 0.72 precision, 0.72 recall and 0.72 F-Measure (FM). Results for individual themes vary, ranging from 0.52 FM (*addiction*) to 0.80 (*Opiates*). Precision is in general higher (>0.70) than recall, except for *Opiates, depression and cripplingalcoholism*.

Finally, Table 5 illustrates the overall accuracy for all four different classifiers. The Mean Reciprocal Rank (MRR) measure weighs in the probability distribution for each prediction and takes into account the position (in ranked order) of the true class in comparison to the actual prediction. More details on this measure are provided in the Methods section. As expected, MRR is consistently higher than accuracy for all classifiers and also shows that posts can transcend multiple themes (see Discussion section).

## Discussion

In our study, we addressed the problem of characterising and automatically classifying user-generated content on the social media platform Reddit for the case of mental health conditions. We manually investigated data originating from several subreddits to group the posts into overarching themes. The derived grouping of posts into themes was further evaluated by applying topic detection algorithms and results suggested that the theme-based grouping was valid. We then applied two classification strategies, a binary classification to determine whether or not a post contains mental health related content, and multiclass classification to identify the mental health condition (theme) a post is referring to. Our results show that by applying a CNN approach in the binary classification task, we achieve an accuracy of 91.08% in distinguising the mental health related posts from unrelated posts. Further to that, we can identify Reddit posts as belonging to one of the 11 defined themes with an overall weighted precision of 0.72 and a recall of 0.71 (0.72 FM). Taken in conjunction, these results suggest that we can reliably identify mental health content and determine the mental health theme that is further referred to in the post, assuming that there is sufficient data for disorders to train the model on.

In our approach, we assessed posts from several subreddits that were grouped into themes. Despite the manual and automated verification of the generated themes, there is an intrinsic overlap of post content across the themes due to the relatedness of mental health conditions. For example, as can be seen from the confusion matrix in Fig 2, a large number of *Opiates* posts are misclassified as *cripplingalcoholism* and vice versa. Both themes refer to a substance addiction, indicating shared language features to a larger degree than with other subreddits. Furthermore, it is worth noting that both subreddits contain strong language that is not as clearly represented in any of the other subreddits, and both subreddits allow for posts that embrace addiction. Similarly, a high rate of posts are misclassified between the *selfharm* and *SuicideWatch* subreddits, both of which feature behaviours associated with mental and emotional distress.

We chose to use topic modelling as a means of automatically obtaining key characteristic words and phrases from the posts, as it has been applied successfully in similar settings in other studies (e.g. Schwartz *et al*. 2016^12^). The topic models (shown in supplement; each topic is using the 10 most descriptive keywords) align with the manually created themes, and an extension of this work could be to further optimise the validation approach by scaling the number of topics retrieved with the number of available posts in the theme.

The control group built for the first task (see section “Control dataset generation” in Methods for more details) utilises the author information of users who post on mental health-related subreddits. We opted for this approach due to the long computation times required for calculating age and gender estimates for individual posts and the number of posts that need to be matched. In general, age and gender matching is desired to avoid influences originating from gender or age differences in language use. While we used all users posting on mental health subreddits, only a subset of authors appears in the control dataset (around 9% of the users; 32,280 appear in the non mental health subreddits and 348,040 appear in the mental health subreddits). Moreover, this intersection of 32,280 users/authors is responsible for 62,513 posts in the mental health subreddits, which corresponds to only 13.6% of the mental health posts (see Supplement, Figure S1 for more details). This may be partially explained by users not wanting to be recognised by other Reddit users or peers and therefore disguising their identity by using so called throwaway accounts^13^. Hence, the results presented here concern cases where authors between the two classes are largely different.

Our study does not focus on such cases, where authors post simultaneously under both classes. It has been demonstrated in the past that characteristic linguistic features are apparent in those suffering from mental illness^12^,^14^,^15^,^16^. This means that our dataset could have limitations in being applicable to assess linguistic differences between mental health and non-mental health related posts, as linguistic features are likely to be present in both the mental health and control posts. With regards to our results, this is likely to increase the difficulty of our classification task, as we are aiming to use the *content* to determine mental health and non-mental health *posts*. On the contrary, if we were to create a control group that is entirely unrelated, it is possible that our performance measures (currently best-performing: 91.08% accuracy for CNN) might increase.

Concerning the classification tasks and the deep learning approaches presented, we experimented with different settings and thresholds. We considered pre-trained word vectors as input to the classifiers (e.g. using Glove’s Common Crawl containing 840 Billion tokens^17^), but the results did not improve. We attribute this to the size of our dataset which is adequate for representing the language within the corpus. Furthermore, we experimented with increasing the complexity of our networks in different parts, such as the dimensions of the input vectors (16), the overall topology of the network, as well as different activation and objective functions. In all the configurations experimented with, the accuracy of the classification did not improve, which is why we kept the simplest configuration.

Our results for the multiclass classification are satisfactory, and provide some interesting insights. There is some variability in class-level results, for instance, for *Opiates*, FM is as high as 0.80, while for *addiction* results are lower (0.52). When studying the confusion matrix (Fig. 2), the most common misclassification is *depression*, for which there are two main explanations. The first reason is that *depression* is the most populous class, so the training of the classifier may have resulted in a bias favouring this class. The second reason is that *depression* is often a secondary symptom of other mental health problems and shows considerable comorbidity with other mental health disorders^18^. This is also confirmed through our manual survey of the content of the posts (see section “Manual characterisation of subreddits”), where it was found that posts from different themes may also concern depression, and where posts in the *depression* subreddit may be related to more than just depression. To further support the above, we conducted an experiment whereby we removed the most prevalent class-theme (*depression*, which accounts for 42% of the posts) and repeated the same multiclass classification task. Contrary to the typical behaviour in such settings, the removal of the most prevalent class resulted in an increase in the accuracy. More specifically, all classifiers improved and CNN performs the best with 79,8% accuracy (more than 8% improvement). This finding further supports the notion that the classes in our problem are neither orthogonal nor mutually exclusive in nature.

When considering a less strict evaluation metric (i.e. instead of True/False as in classification tasks) such as Mean Reciprocal Rank (i.e. the position at which a theme ranked compared to the actual class), the results improve significantly (more than 12% across all 4 classifiers). This finding also highlights the fact that some of the themes are highly inter-related and not always distinguishable as separate and exclusive classes. For example, a common symptom of depression is suicidal ideation, which in consequence means that a person posting on *depression* may express suicidal thoughts, which are the content of communications on *SuicideWatch*.

Concerning the binary classification task, we notice that the simple FF network achieves similar performance to the more sophisticated CNN (less than 0.3% drop in accuracy), concluding that the binary classification problem is captured sufficiently using a more simple neural network.

One limitation of our approach is the number of mental health themes that are used in the multiclass classification task. In order to cover more themes, our model built for the multiclass classification would require additional data. However, the way the approach is implemented, more themes can be added over time and the only cost is that the prediction model needs to be retrained and performance re-assessed.

In conclusion, our suggested method is applicable to the identification of posts relevant to a mental health subreddit as well as the identification of the actual mental health theme they relate to. Being able to classify posts in this manner is the first step in the direction of targeted interventions, e.g. by redirecting posts that seem in need of urgent moderator attention. In future work, we aim to further improve the classification into mental health themes (multiclass classification), address inter-related themes e.g. by employing hierarchical structures, increase the coverage of mental health themes and devise an “urgency scheme” to come closer to the aforementioned notification system in the frame of real-time, personalised interventions.

## Methods

### Storage and indexing of complete collection of Reddit posts

For the purpose of this study, we downloaded a dataset from https://redd.it/3mg812 that contained all the posts made to Reddit (independent from subreddits) from 01.01.2006 to 31.08.2015. This dataset is publicly available and was generated by a Reddit user through accessing the Reddit Application Programming Interface (https://www.reddit.com/dev/api). In order to discover the relevant subreddits, we used the Elasticsearch (https://www.elastic.co/) search engine to index the complete dataset. Our analysis reports on aggregated data only, which adheres to Reddit’s published terms and conditions.

### Discovery of subreddits

As of November 2016, there are over 900,000 subreddits (http://redditmetrics.com/history). Each subreddit invites users to participate in a discussion concentrated around a specific topic. The topics can vary, from a general to a very specific topic. For instance, e.g. the subreddit Ask Me Anything (AMA) is about a single person answering questions posed by the public and has around 140,000 subscribers. Similarly, the subreddit Playstation invites users around the popular game console and has 36,000 subscribers. In order to identify the subreddits that are related to mental health, we adopted a semi-supervised approach. The first step was to leverage on a pre-existing keyword list curated by domain experts, for the purpose of identifying social media content linked to mental health^19^. From the obtained candidate list of subreddits, we assessed the subreddits with a high number of posts and manually selected those that corresponded to the conditions of interest for this study. All relevant subreddits and their descriptions are provided in the Supplementary material.

### Manual assessment and selection of subreddits

To gain a deeper understanding of the nature of posts on these subreddits, we conducted a manual characterisation of posts contained in the selected subreddits. For this purpose,10 random posts (including their title) were collected from each of the subreddits. Two of the authors of this manuscript (AO, SV) independently read through these posts and collected notes as to whether the post was relevant to the condition the subreddit was selected from, the post concerned the poster or someone else and what the type of the post was (request for help, engaging, sharing story, etc.). A joint discussion was held to finalise the set of subreddits and themes to use for classification and identify potential differences in interpretation.

### Control dataset generation

In order to identify Reddit posts concerning mental health, we generated a control dataset that is non-mental health related. The first step included the collection of all authors from our mental health dataset together with the date of their first (index) posting in any one of the mental health subreddits. For each author-date pair, we retrieved all of their posts across any other subreddit and defined these as non-mental health related. In order to avoid time overlap in the generation of the control dataset, we only kept non-mental health posts by authors that were at least 180 days older than their index post in a mental health subreddit. The 180 day window was chosen both to ensure a time clinically distant from the mental state at the index post of the mental health subreddit, and to result in a balanced dataset.

### Topic detection

As the manual characterisation only covered a very small proportion of posts, we aimed to verify the grouping of the posts by building topic models across the themes. Topic models identify groups of words that are related to each other and therefore constitute a *topic*, presented in textual data. Here, we used the Latent Dirichlet Allocation implementation as provided by the Python package *gensim*^20^ while setting the number of topics to 10 (*n* = 10) and rest of the parameters to default. Data from each of the relevant themes was handled and processed individually. For this purpose, if a theme contained more than one subreddit, all the posts of the relevant subreddits were merged together before processing. We removed English language stop words as indicated in NLTK^21^ (frequently used words, such as “and” and “the”, that are expected to carry little characterising information; see supplement for more information) from all the posts before determining topics.

The underlying assumption when using topic models in this case is that a grouping of subreddits in cohesive groups will result in topic models that are relevant to aspects of the corresponding theme (e.g. symptoms or treatments). While topic modelling can be used for validating the grouping of subreddits, it possesses the added benefit of providing insights into the contents discussed on the subreddits investigated.

### Evaluation metrics

In our evaluation, we used the following metrics:


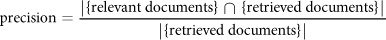



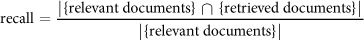



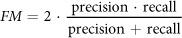



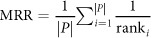


where *P* is the set of classified posts and rank_*i*_ refers to the rank position of the relevant theme for the *i*-th post.

### Classification of Reddit posts

We considered two different classification problems. The first concerns the binary classification of posts as being relevant to mental health or not. The second classification problem deals with the multiclass classification of the 11 mental health themes resulting from the manual and topic assessment steps. For both tasks, we applied the same methodology and evaluation approach, which includes training four different classifiers (FF, CNN, Linear regression, SVM, explained below).

The first step towards classifying the Reddit posts was to preprocess the corpus. This step included the concatenation of the post title with the body of the post. We then transformed all text into lower case. Afterwards, we prepared the text to be represented as Word Embeddings^22^ within the Neural Network. This was achieved by creating a word dictionary from the corpus itself, for which we kept the 5,000 most frequent words (including stopwords, which accounts for 99.999% of the total number words). The last step was to replace all words with their indices, so as to represent each post as an array of integers. For the words that were not frequent enough (lower than 0.001% of the total number of words), a single, unique integer was assigned. Finally, we only kept the first 300 words/integers for each post.

**Figure 3:**
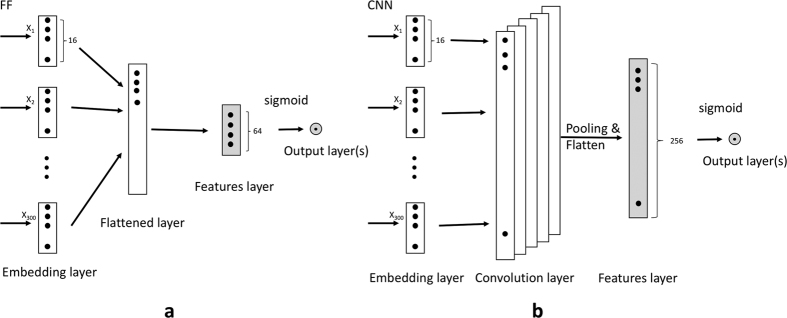
Architecture for the Feed Forward (**a**) and Convolutional Neural Network (**b**) deep learning approaches.

The first two classifiers are based on a Neural Network (NN) architecture. Both include the same number of layers and are trained with a similar approach. The first classifier is a Feed Forward NN (see Fig. 3a). The second classifier implements a Convolutional NN, with a filter window of 3 (see Fig. 3b). The first layer takes as input the array of integers representing the content and implements an Embedding layer where each word vector has a dimension of 16. The intermediate layer is implemented using a dense structure with 64 (FF) and 256 (CNN) neurons that are used to represent the feature space. The final layer contains the number of output nodes needed for the classification task (i.e. 2 for the binary and 11 for the multiclass classification).

As shown in Fig. 3, the first layer in both networks is an Embedding layer that turns an array of integers into a vector of size 16 (word embeddings). For the CNN, we added an extra, convolutional layer with a 5-length filter and max pooling with length 2. For the FF, the equivalent was a flattened layer. The next step for both networks is a dropout filter of value 0.25 (to avoid overfitting) and a dense layer of 64 and 256 neurons for the FF and CNN, respectively. We used rectified linear unit (ReLU) as activation function and the objective function was categorical cross entropy. For the training process, we set a maximum of 100 epochs with a stopping criterion of 5 epochs for no increase in the objective function. For both networks and classification problems, training stopped before 25 epochs had been reached.

We also considered two more classification approaches as a baseline. Both use the same input as the NNs described above but implement a linear regression and an SVM-based classification approach. We consider these to be our baselines. The details for these approaches are provided in the supplement. All four classification approaches were trained using the same training-test split (80/20) on the dataset and the same preprocessing steps.

## Additional Information

**How to cite this article**: Gkotsis, G. *et al*. Characterisation of mental health conditions in social media using Informed Deep Learning. *Sci. Rep.* **7**, 45141; doi: 10.1038/srep45141 (2017).

**Publisher's note:** Springer Nature remains neutral with regard to jurisdictional claims in published maps and institutional affiliations.

## Supplementary Material

Supplementary Information

## Figures and Tables

**Table 1:** Summary and description of the mental health condition themes and their originating subreddits used in this study.

Theme	#Posts	Description
BPD	11,880	Forum to discuss aspects of Borderline Personality Disorder either as a sufferer, someone closely related to a sufferer, or someone interested in this disorder
bipolar (*BipolarSOs, BipolarReddit, bipolar*)	41,636	Communities to discuss issues surrounding Bipolar Disorder; while bipolar and BipolarReddit focus on sufferers and their support, BipolarSOs invites contributions from people that are in a relationship with someone suffering from Bipolar Disorder
schizophrenia	4,963	Subreddit to discuss schizophrenia-type disorders and schizophrenia-related issues such as psychosis
Anxiety	57,523	Forum for anything that is related to an anxiety disorder; does not distinguish between sufferer or someone related to a sufferer
depression	197,436	A community for helping anyone struggling with depression; posters are not limited to those who have received a diagnosis by their GP/hospital doctor and the emphasis is on supporting others in their struggle with depression
selfharm (*selfharm, StopSelfHarm*)	17,102	Forums to discuss aspects of people self-harming; while selfharm aims to build a community of sufferers, StopSelfHarm focusses on supporting anyone wanting to stop self-harming even if through a related person
SuicideWatch	90,518	Forum to support individuals thinking about suicide or people thinking of someone else being at risk of suicide
addiction	4,360	Community to discuss any physical or psychological dependence, e.g. drugs or video games; encourages self post, but does not exclude non-sufferers
cripplingalcoholism	38,241	Community for alcohol-dependent people, with an emphasis on the acceptance of the condition, also stretching to embracing their condition
Opiates (*OpiatesRecovery, opiates*)	65,143	Forums to discuss opiate addiction; while opiates addresses all aspects of the addiction, OpiatesRecovery focusses strongly on supporting everyone wanting to withdraw from opiates; Posting to opiates is restricted to people aged over 18 years
autism	9,470	Forum for anything related to an Autism Spectrum Disorder; provides information and support to anyone facing a diagnosis whether for themselves or someone else
Non-mental health	476,388	Control dataset generated using posts from users on subreddits outlined above, who have posted on other subreddits that are not mental health related

Where appropriate, multiple subreddits participating in one theme are presented in brackets.

**Table 2:** Evaluation results for the binary classification of mental health posts using all classifiers.

	FF	CNN	Linear	SVM
Precision	92.05%	91.76%	87.31%	88.50%
Recall	88.83%	89.83%	83.57%	81.75%
FM	0.90	0.91	0.85	0.85
Accuracy	90.78%	91.08%	86.01%	85.87%

FF = Feed Forward, CNN = Convolutional Neural Network, SVM = Support Vector Machine. FM = F-measure (harmonic mean of precision and recall).

**Table 3:** Confusion matrix for the binary classification of mental from non-mental health content using a Convolutional Neural Network classifier.

	Non-mental	Mental
Non-mental	87821	7361
Mental	9277	81986

Results concern the test dataset (20% posts held out from training). Rows = actual labels, Columns = predicted labels.

**Table 4:** Multiclass classification evaluation metrics using a Convolutional Neural Network.

Theme	Precision	Recall	FM
BPD	0.88	0.46	0.60
bipolar	0.77	0.60	0.67
schizophrenia	0.75	0.48	0.58
Anxiety	0.83	0.75	0.79
depression	0.70	0.77	0.73
selfharm	0.70	0.58	0.64
suicidewatch	0.62	0.59	0.61
addiction	0.72	0.41	0.52
cripplingalcoholism	0.68	0.76	0.72
Opiates	0.76	0.86	0.80
autism	0.84	0.71	0.77
Weighted average	0.72	0.71	0.72

**Table 5:** Overall evaluation results for four different classification approaches.

	FF	CNN	Linear	SVM
Accuracy	70.82%	71.37%	58.72%	64.02%
MRR	0.82	0.83	0.74	0.78

FF = Feed Forward, CNN = Convolutional Neural Network, SVM = Support Vector Machine. MRR = Mean Reciprocal Rank.
