# Corrigendum: Characterisation of mental health conditions in social media using Informed Deep Learning

**DOI:** 10.1038/srep46813

**Published:** 2017-05-16

**Authors:** George Gkotsis, Anika Oellrich, Sumithra Velupillai, Maria Liakata, Tim J. P. Hubbard, Richard J. B. Dobson, Rina Dutta

Scientific Reports 7: Article number: 4514110.1038/srep45141; published online: 03 22 2017; updated: 05 16 2017

One of the Supplementary Information files that accompany this study was inadvertently omitted in the original version of this Article. The link to this Supplementary Information file has now been added to the HTML version of the Article.

