# Supplementary material for: Characterisation of mental health conditions in social media using Informed Deep Learning
Source: Sci Rep. 2017 Mar 22;7:45141. doi: 10.1038/srep45141 (PMC5361083; doi:10.1038/srep45141)
Supplement: Supplementary Information [file srep45141-s1.pdf]

Characterisation of mental health conditions in social media using Informed Deep Learning [SUPPLEMENT].

George Gkotsis[1,*], Anika Oellrich[1], Sumithra Velupillai[1,2], Maria Liakata[3], Tim JP Hubbard[4], Richard JB Dobson[1,5], and Rina Dutta[1]

[1]King's College London, IoPPN, London, SE5 8AF, UK
[2]School of Computer Science and Communication, KTH, Stockholm
[3]Department of Computer Science, University of Warwick, Coventry
[4]King's College London, Department of Medical & Molecular Genetics, London, SE1 9RT
[5]Farr Institute of Health Informatics Research, UCL Institute of Health Informatics, University College London, London, WC1E 6BT, UK
[*]george.gkotsis@kcl.ac.uk

ABSTRACT

This document is a supplement to the main manuscript. It provides further details behind our methodology and results.

Introduction

This document provides additional information on results and methods that have not been provided as part of the main manuscript. This information concerns mainly the classification methods of Reddit posts both as a binary classification task (i.e. whether a post is related to mental health) and as a multiclass classification task (i.e. which mental health theme a post belongs to). We note that this document relies on information provided in the main text, which is why we advise the reader to first read through the main manuscript.

Discovery of subreddits

In order to determine subreddits relevant to our study, we used an expert-curated keyword list (see Table S1) that was generated as part of the Pheme project[1]. We then identified posts using these as keywords in queries and recorded the subreddit they belong to. From these search results, an initial list with the most prevalent subreddits was considered. A follow-up manual selection resulted in a list of 16 for further investigations. Table S2 provides additional information for the 16 selected subreddits.

anxiety*, autism*, Abilify, Abixa, ACP103, ACP-103, ACP103, Akatinol, alzheimer*, Amazeo, Amidone, Amipride, amisulpride, Amival, Aricept, aripiprazole, Ativan, Atosil, Avanza, Avomine, Axit, Axura, brexpiprazole, Brintellix, Buprenex, buprenorphine, Butrans, cariprazine, Celexa, CibalithS, Cipramil, citalopram, Clopine, clozapine, Clozaril, Convulex, dementia, depress*, Diastat, DiastatAcuDial, diazepam, Dolophine, donepezil, Ebixa, Effexor, Epilim, Episenta, Epival, Eskalith, Exelon, Fargan, Farganesse, FazaClo, fluoxetine, galantamine, Haldol, haloperidol, Heptadon, Imovane, Invega, Lanzek, Latuda, Lergigan, Lithobid, lorazepam, lurasidone, Lustral, Lycoremine, memantine, Memox, methadone, Methadose, Mirtaz, mirtazapine, Mirtazon, Namenda, Nivalin, Nuplazid, obsessivecompulsive, ocd, OCP34712, olanzapine, OPC34712, OPC-34712, paliperidone, Phenergan, Physeptone, pimavanserin, promethazine, Promethegan, Prothiazine, Prozac, quetiapine, Razadyne, Receptozine, Remeron, Reminyl, RGH188, RGH-188, RGH188, Risperdal, risperidone, rivastigmine, Romergan, Sarafem, schizoaffective, schizoaffective, schizophren*, selfharm*, selharm*, Seroquel, sertraline, Solian, Soltus, Sominex, Subutex, suicid*, Sulpitac, Sulprix, Symoron, Trintellix, Valium, valproate, venlafaxine, Versacloz, vortioxetine, Zaponex, Zetran, Zimovane, Zispin, Zoloft, zopiclone, Zypadhera, Zyprexa

Table S1. List of keywords used for the discovery of relevant subreddits. Asterisks (*) were used to retrieve inflations of the target-word. From Gkotsis et al.[2].

Control dataset generation

Figure S1 illustrates the way the authors (users) in our dataset contribute towards the generation of our datasets. It also shows that the authors used for the generation of the control dataset contributed a small portion of mental health related posts.

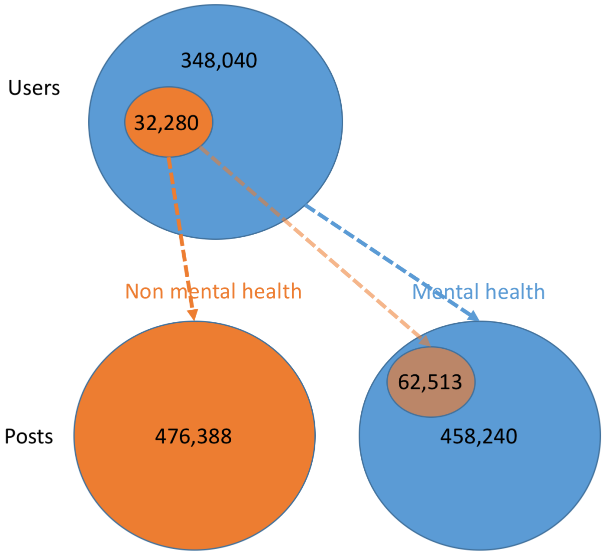

Figure S1. A visualisation of the users in our study and their contributions to the posts dataset. 348,040 users authored a post under a mental health related theme. 32,280 of them posted elsewhere under a non-mental health subreddit (control dataset). At the same time, these authors are only partially responsible for the generation of the mental health posts (i.e. just 62,513 posts compared to the 458,240 total number of mental health posts).

In descending order, the 10 most prevalent non-mental health sureddits are:

- AskReddit
- trees
- leagueoflegends
- circlejerk
- reddit.com
- gaming
- askscience
- buildapc
- atheism
- explainlikeimfive

A complete list with the number of posts for each of the non-mental health sureddits is provided online[1].

Figure S2 illustrates the number of authors found in each of the themes of our study (left-to-right diagonal). For the remaining of the cells, the cells represent the number of authors who have published at least one post in pairs of themes.

[1] https://github.com/gkotsis/reddit-classification/blob/master/subreddits.xlsx

Classification of mental health content

For the classification task, as already discussed in the main manuscript, we considered four different classifiers. The approach for the deep learning (neural network) based classifiers (i.e. Feed Forward and Convolutional Neural Network) is presented in the main manuscript. For the *linear-based* classification, we used a multinomial logistic regression approach, where posts are analysed as 2-grams and these 2-grams are being represented as bag-of-words. In our approach, the discrete features are word counts. For the *SVM-based* classification, we first applied a tf-idf (term frequency-inverse document frequency) representation of the words in the posts. We then applied a stochastic gradient descent algorithm implementing a regularized linear model[3].

The process of classifying Reddit posts was separated into two individual tasks, which play a role in the future application of the classification results. The first task was a classification of posts into whether or not they are related to mental health issues, further referred to as binary classification. The second task was the classification according to the manually defined and automatically evaluated themes, to determine the mental health condition the post belongs to and possibly the nature of the post. In the following, we provide further information on results achieved, complementing those reported in the main manuscript.

Binary classification of mental from non-mental health related content

The confusion matrices for each of the classifiers are provided in Tables S3 - S6. Across all classifiers, non-mental health related posts are more often misclassified as mental health related than vice versa.

To provide additional information on classifier performance, we also show the Receiver Operating Characteristics, determined based on the measures described in the main manuscript (see Figure S3). The plot shows that CNN performs best, closely followed by SVM and FF on our dataset. Linear results in poorest performance for distinguishing non-mental health and mental health related posts.

Multiclass classification of mental health themes

In the following figures (S4, S5 and S6), we present the confusion matrices for the remaining three classifiers and the multiclass classification task. For all three matrices, the sums on their diagonals are lower than the CNN classifier (presented in the main manuscript). Moreover, we notice that - as a result of what we believe can be attributed to the training process - both SVM and Linear classifiers achieve good results for the most prevalent theme (depression). However, this training bias results in higher loss in the overall classification task, since both FF and CNN (deep learning based) classifiers have better overall performance.

We conducted the following experiment: If a prediction is made on the most prominent theme (depression), we also consider the second highest value as another chance for prediction (Table S7, first column). Afterwards, we repeated the same experiment where we allowed two chances for each prediction (Table S7, second column).

Subreddit	Description	URL
Anxiety	Posters on this subreddit are likely to be anxiety sufferers, though there are no specific intensions set for this subreddit.	https://www.reddit.com/r/Anxiety/
BPD	A place for those who have Borderline Personality Disorder, their family members and friends, and anyone else who is interested in learning more about it.	https://www.reddit.com/r/BPD
BipolarReddit	Aims to be a community of Bipolar sufferers, as opposed to Bipolar-SOs.	https://www.reddit.com/r/BipolarReddit/
BipolarSOs	Community that is composed of people either being affected themselves and/or are in a relationship with someone being affected by Bipolar Disorder.	https://www.reddit.com/r/BipolarSOs/
OpiatesRecovery	Provides support and advise to everyone trying to overcome opiate addiction by others in similar situations.	https://www.reddit.com/r/OpiatesRecovery/
StopSelfHarm	A lot of teenagers and adults suffer with self-harm issues, or consider using self-harm as a way of coping with their struggles. Self-harming can be a very difficult habit to break, if you know someone or you struggle with self-harm get help. You are not alone.	https://www.reddit.com/r/StopSelfHarm
addiction	No description provided	https://www.reddit.com/r/addiction
autism	Autism news, information and support. Please feel free to submit articles to enhance the knowledge, acceptance, understanding and research of Autism and ASD.	https://www.reddit.com/r/autism
bipolar	A safe haven for bipolar related issues. We are a community here not just a help page. Be a part of something that cares about who you are.* /r/bipolar Feel free to post, discuss or just lurk. There is no judgement in this place, we are here for each other.	https://www.reddit.com/r/bipolar
crippling-alcoholism	Provides an exchange board for people that are addicted to alcohol, but primarily those that consider alcoholism a life-style choice.	https://www.reddit.com/r/cripplingalcoholism
depression	A safe, supportive space for anyone struggling with depression.	https://www.reddit.com/r/depression
opiates	No description provided.	https://www.reddit.com/r/opiates
schizophrenia	This is a community meant for a discussion of Schizophrenia and schizophrenia related issues (including psychotic symptoms in general, Schizoid, Schizotypal, and Paranoid Personality Disorders).	https://www.reddit.com/r/schizophrenia
selfharm	A subreddit for self-harmers to relate to each other, ask questions, and build up a community. Giving instructions on methods of self-harm is not allowed on this subreddit.	https://www.reddit.com/r/selfharm
SuicideWatch	No description provided. Guidelines are very strict and thorough.	https://www.reddit.com/r/SuicideWatch

Table S2. The subreddits used in our study, together with a short description as retrieved from their website. From Gkotsis et al.[2].

	Non-mental	Mental
Non-mental	87685	7497
Mental	9729	81534

Table S3. Confusion matrix for the Feed Forward Neural Network and the binary classification task. Rows = actual labels, Columns = predicted labels.

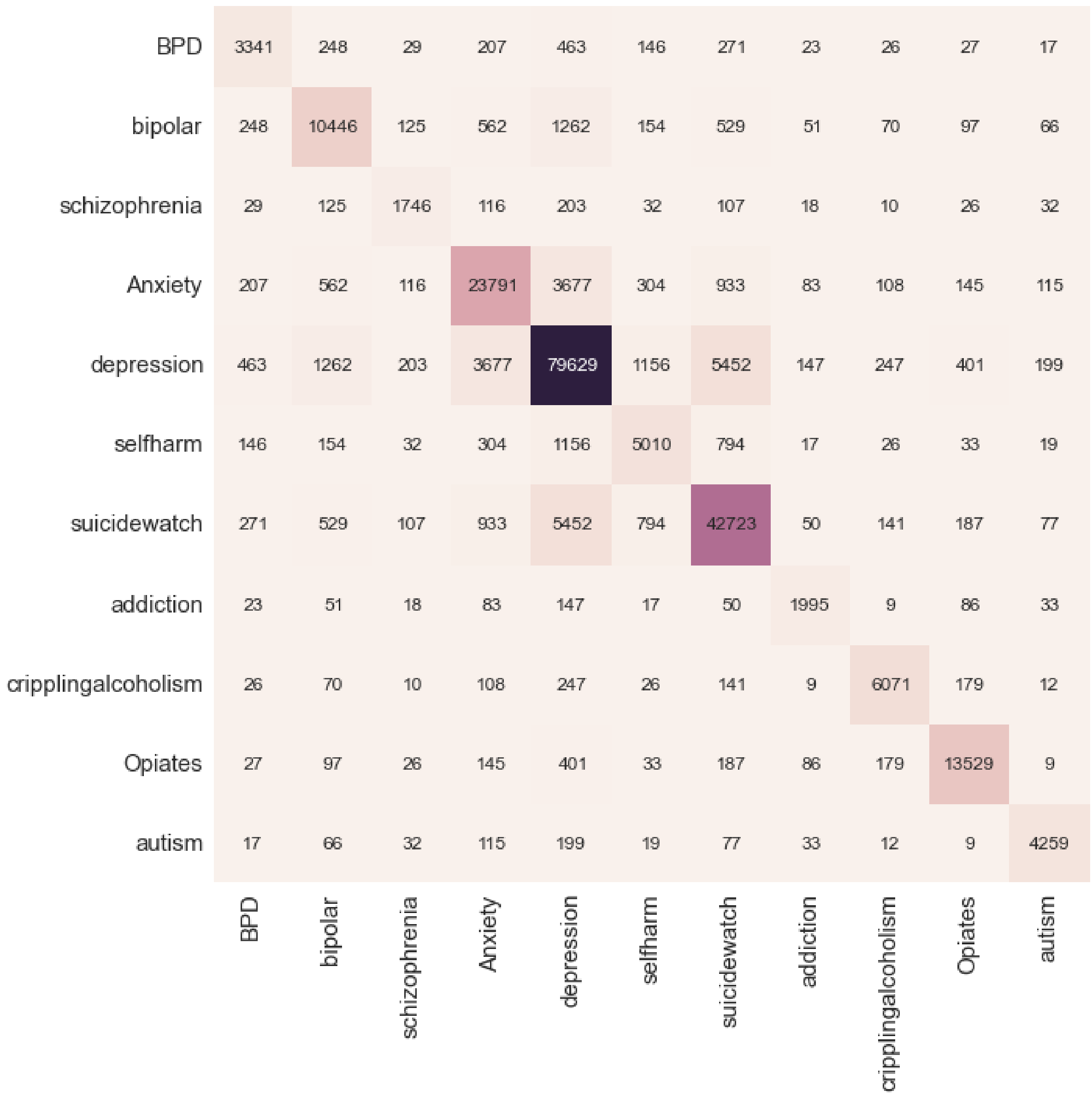

Figure S2. Visualisation of numbers of authors contributing in pairs of subreddits. The left to right diagonal represents the unique authors for each theme. The remainder of the cells represent the number of users appearing in both of the corresponding subreddits.

	Non-mental	Mental
Non-mental	87821	7361
Mental	9277	81986

Table S4. Confusion matrix for the Convolutional Neural Network and the binary classification task. Rows = actual labels, Columns = predicted labels.

	Non-mental	Mental
Non-mental	85459	9723
Mental	16680	74583

Table S5. Confusion matrix for the SVM-based classifier and the binary classification task. Rows = actual labels, Columns = predicted labels.

	Non-mental	Mental
Non-mental	84129	11053
Mental	14981	76282

Table S6. Confusion matrix for the linear classifier and the binary classification task. Rows = actual labels, Columns = predicted labels.

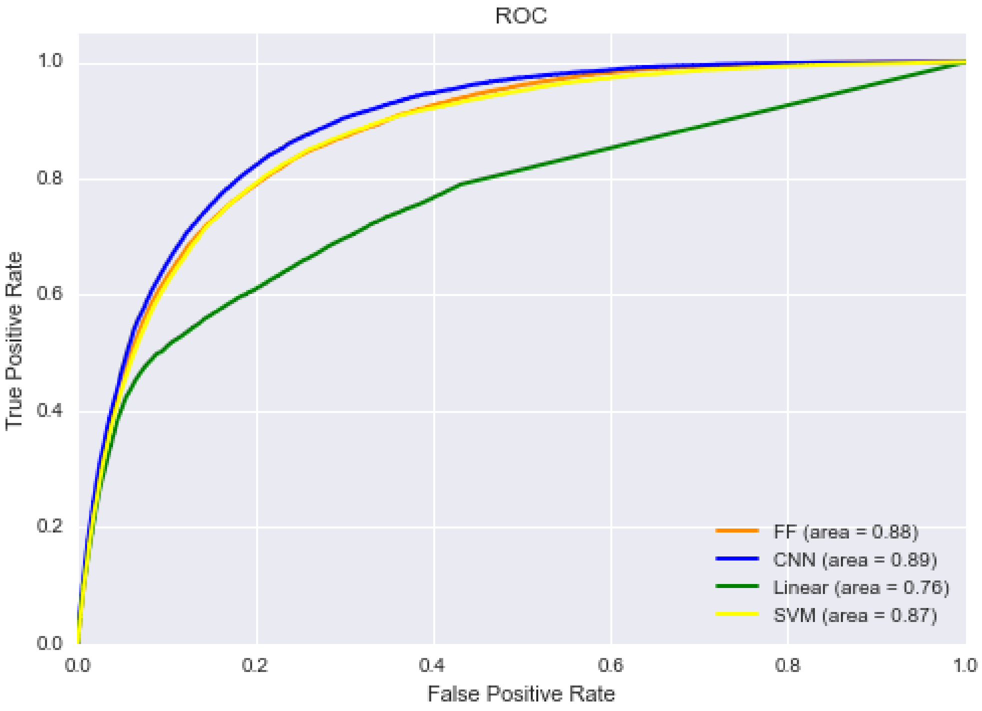

Figure S3. Receiver Operating Characteristics (ROC) for all four different classifiers for the binary classification problem (mental health-related post vs. non mental health-related. FF = Feed Forward, CNN = Convolutional Neural Network, SVM = Support Vector Machine.

	Accuracy (depression)	Accuracy (all classes)
FF	77.63%	87.94%
CNN	78.04%	88.31%
SVM	79.54%	84.74%
Linear	72.06%	78.20%

Table S7. Accuracy results when also considering the second highest prediction from our classifiers. Left column shows results when testing for the second-best prediction only for cases where depression is predicted first. Right column considers all second-best predictions regardless of the first prediction.

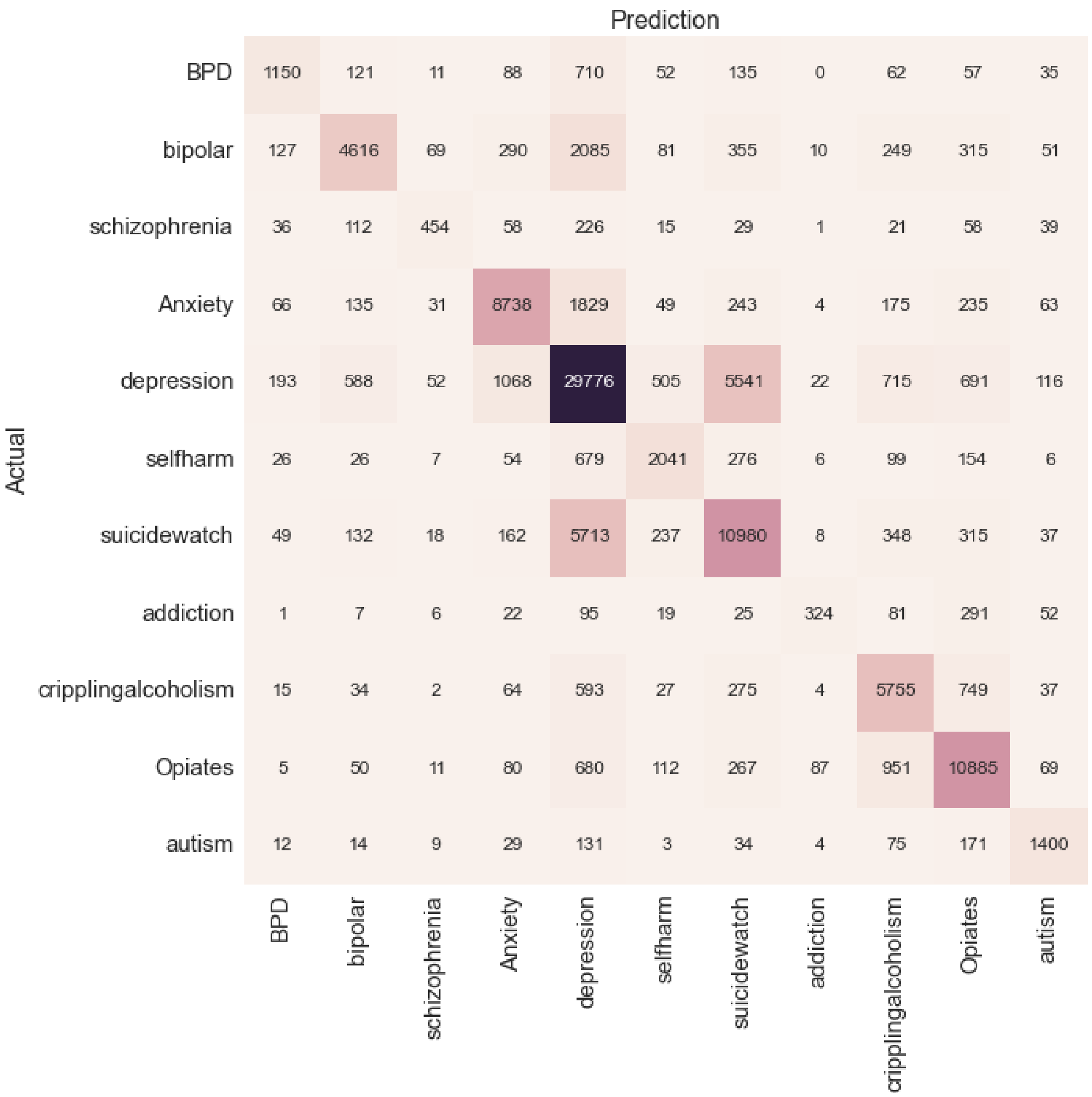

Figure S4. Confusion matrix for the Feed Forward Neural Network and the multiclass classifcation task.

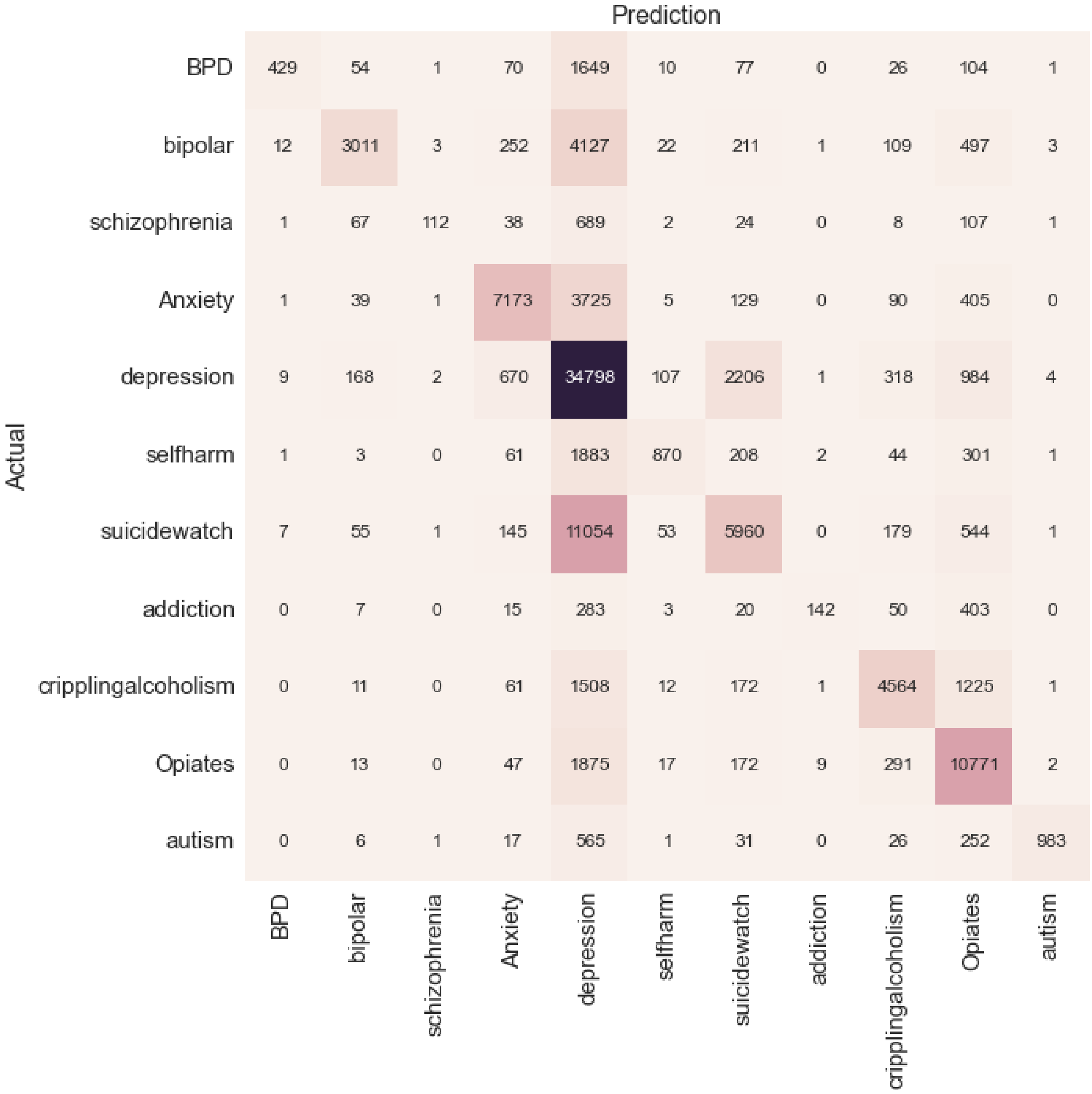

Figure S5. Confusion matrix for the SVM-based classifier and the multiclass classifcation task.

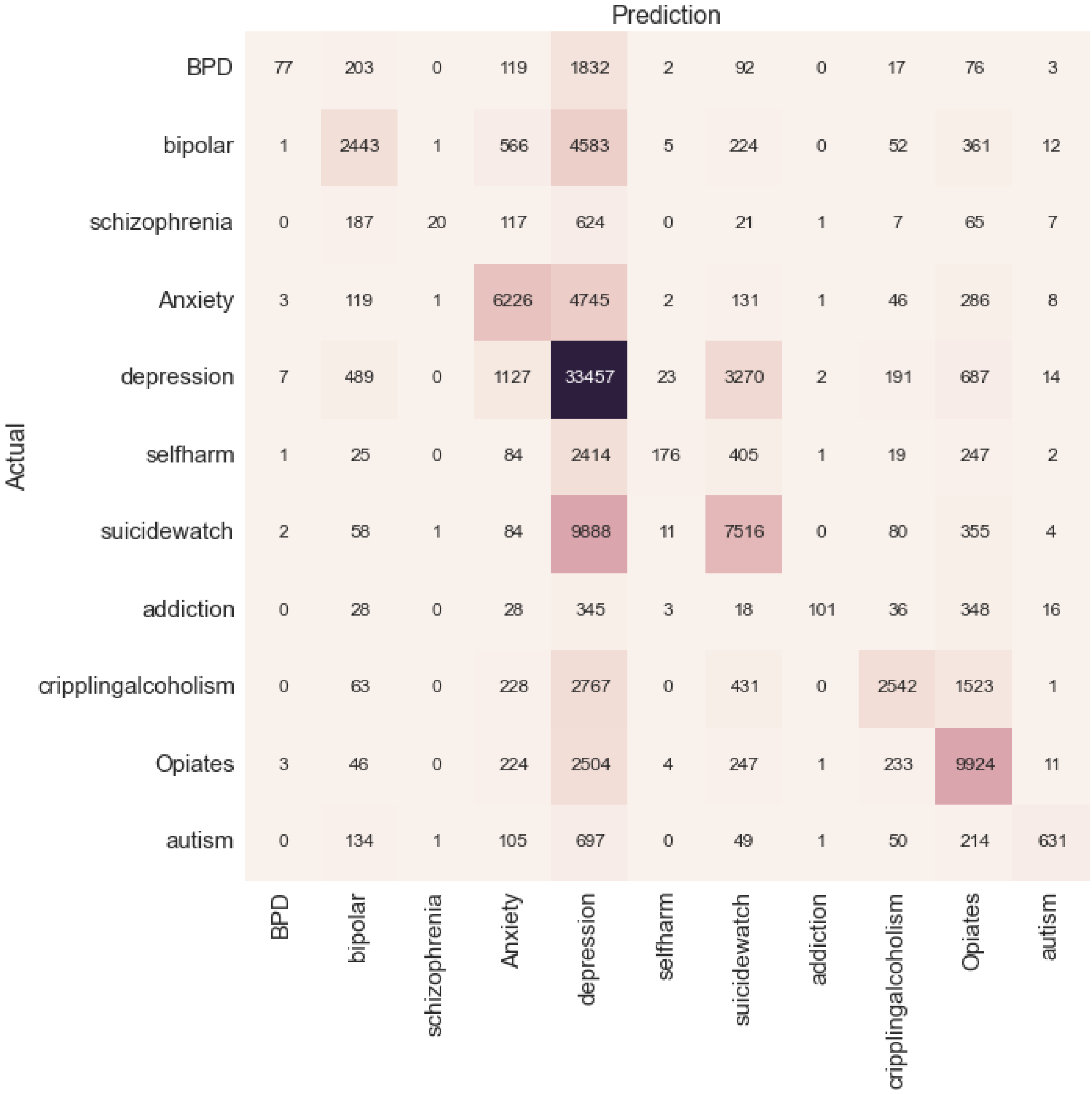

Figure S6. Confusion matrix for the linear classifier and the multiclass classifcation task.

References

1. Kolliakou, A. *et al.* D7.2.2.2 - Annotated Corpus - Final Version Public Deliverable, PHEME Project (FP7-ICT-611233) (2015).

2. Gkotsis, G. *et al.* The language of mental health problems in social media. In *Proceedings of the 3rd Workshop on Computational Linguistics and Clinical Psychology: From Linguistic Signal to Clinical Reality*, 63–73 (2016).

3. Shalev-Shwartz, S., Singer, Y., Srebro, N. & Cotter, A. Pegasos: Primal estimated sub-gradient solver for svm. *Mathematical programming* **127**, 3–30 (2011).
